# The evidence-based COPE program: Reducing the time between diagnosing and treating depression and anxiety in youth

**DOI:** 10.1097/01.NPR.0000000000000152

**Published:** 2024-02-22

**Authors:** Bernadette Mazurek Melnyk

**Affiliations:** **Bernadette Mazurek Melnyk** is creator of the COPE program and founder of COPE2Thrive, LLC. She is a globally recognized expert, speaker, author, and researcher in the areas of evidence-based practice, mental health and well-being, and intervention research.

**Keywords:** adolescents, anxiety, children, COPE, COPE program, depression, evidence-based intervention, young adults

## Abstract

The soaring prevalence of depression and anxiety in children, teenagers, and young adults is now a public health epidemic, yet access to timely evidence-based mental health treatment is often lacking due to a severe shortage of mental health providers. This article provides an overview of the current state of depression and anxiety in children and adolescents as well as first-line evidence-based treatment. The Creating Opportunities for Personal Empowerment (COPE) program, a cognitive-behavioral skills-building intervention, is highlighted as an evidence-based intervention for timely treatment that can be delivered by NPs, physicians, and physician associates/assistants in primary care settings, school-based health centers, and chronic care clinics with reimbursement as well as in schools and universities as a preventive mental health intervention.

## Abstract 2

The soaring prevalence of depression and anxiety in children, teenagers, and young adults is now a public health epidemic, yet access to timely evidence-based mental health treatment is often lacking due to a severe shortage of mental health providers. This article provides an overview of the current state of depression and anxiety in youth as well as first-line treatment. The Creating Opportunities for Personal Empowerment (COPE) program, a cognitive-behavioral skills-building intervention, is an evidence-based intervention for timely treatment that can be delivered by NPs, physicians, and physician associates/assistants in various settings as a preventive mental health intervention.

**Figure:**
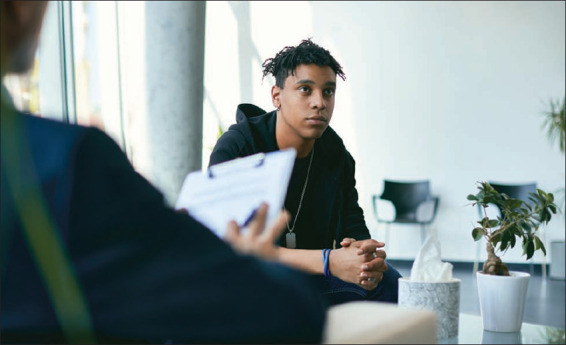
No caption available.

Even before the COVID-19 pandemic, mental health problems in children, adolescents, and young adults were a growing public health concern.^1^ In the wake of the pandemic, mental health disorders skyrocketed among children and adolescents, reaching epidemic proportions with a prevalence of 25.2% for depression and 20.5% for anxiety globally.^2^,^3^ In a recent analysis of 32 billion private healthcare claims for people ages 13 to 18 years, findings indicate an increase of 93.6% in generalized anxiety disorder (GAD) and 83.9% in major depressive disorder (MDD) from April 2019 to April 2020.^4^ For those ages 19 to 22 years, GAD increased by 67.5% and MDD by 49.6%. In a systematic review of pediatric mental health problems during the COVID-19 pandemic, findings revealed other adverse mental health outcomes, including worry, helplessness, fear, nervousness, agitation, and aggression.^2^ Additionally, some studies in this systematic review reported heightened emotional symptoms, conduct problems, hyperactivity-inattention, and peer problems, as well as a decrease in prosocial behaviors.

Suicide is the second leading cause of death among individuals ages 10 to 14 and 20 to 34 years in the US.^5^ However, in Colorado specifically, suicide recently rose to the number one cause of death in individuals ages 10 to 19 years, resulting in a public health emergency declaration.^6^ In the US, ED visits for suicide attempts rose 51% for adolescent girls in early 2021 as compared with the same period in 2019.^7^ Amid the pandemic, one in four young adults considered suicide.^8^ Although depression is a major risk factor for and anxiety is associated with suicide, these two conditions are often undiagnosed or untreated.^9^ In response to the youth mental health crisis, the US Surgeon General issued a public health advisory in 2021, calling the American people's attention to the challenge and recommending urgent action.^1^

A major barrier to addressing the mental health needs of children and youth is the ongoing deficit of accessible mental health services.^10^ The chronic shortage and geographic maldistribution of mental health professionals have been key drivers in the surge of unmet needs in mental health care. Prior to the pandemic, just half of US children with a treatable mental health disorder received needed care or counseling.^11^ Most states have fewer than 40% of the mental health care professionals required to meet the needs of their population, leaving over 72% of the nation with unmet mental health care needs.^10^ Over 122 million individuals live in federally designated health professional shortage areas for mental health care, with rural populations experiencing the greatest disparity in access to mental health providers for prevention and treatment.^12^,^13^

## Time gap between diagnosis and treatment

The US Preventive Services Task Force (USPSTF) recommends screening for MDD in people ages 12 to 18 years.^14^ Further, it advises that screening should be implemented with adequate systems in place to ensure accurate diagnosis, effective treatment, and appropriate follow-up. The USPSTF also recommends screening for anxiety in children ages 8 to 18 years.^15^ However, many healthcare providers across the country have yet to implement these screening recommendations consistently due to the shortage of mental health care providers for referral and inadequate knowledge regarding how to obtain reimbursement for screening.^16^ Even with routine screening and diagnosis of depression and anxiety in primary care settings, patients in the pediatric and young adult populations often face extensive wait times (2 to 3 months) for needed mental health treatment due to mental health care professional shortages.^17^ Unfortunately, the average time gap between onset of a mental health issue and commencement of treatment is 11 years.^18^ Significant racial/ethnic disparities also exist in receiving mental health services, with fewer Black and Hispanic children and teenagers receiving treatment than White children and teenagers.^19^,^20^

## First-line treatment for depression and anxiety

Depression and anxiety have multiple causes, including biological changes in the chemistry of the brain (for example, imbalances in serotonin, dopamine, or norepinephrine or excess cortisol); genetic influences; environmental causes (for example, stressful situations); adverse childhood events; physical health disorders; and depressogenic cognition (defined as an unhelpful or negative pattern of automatic thoughts). Examples of negative automatic thoughts include labeling (for example, “I'm stupid” or “I can't do anything right”), catastrophic thinking (for example, “I got a D on a final exam; I'm going to fail this course and flunk out of school”), and mindreading (for example, “they think I'm ugly” or “I didn't get an invitation to the party because they don't like me”). Unless these types of automatic unhelpful or negative thoughts are “caught” or recognized when they occur, checked for helpfulness or truth, and changed to more positive thoughts, they can result in depression, anxiety, stress, or anger.

A strong body of research exists to support the efficacy of cognitive behavioral therapy (CBT) in reducing depression and anxiety in children, teenagers, young adults, and adults.^17^ CBT is based on the cognitive theory of depression developed by Aaron Beck and behavioral theories developed by Skinner and Lewinsohn.^13^,^21^-^24^ A negative cognitive triad consists of a negative view of oneself, one's environment, and the future. Cognitive theory focuses on becoming aware of or catching one's cognitive automatic distortions or unhelpful negative thoughts or beliefs, checking them by asking whether the thoughts are helpful or true, and changing them into positive ones to feel emotionally better and engage in healthy behaviors (that is, “catch, check, and change”). This is often referred to as the thinking, feeling, and behaving triangle (that is, how we think affects how we feel and behave). Negative or unhelpful patterns of thinking lead to anxiety, depression, and hopelessness.

Active CBT consists of reducing negative thoughts (cognitive restructuring), increasing pleasurable activities (behavioral activation), and improving assertiveness and problem-solving skills (homework assignments/skill-building activities). The American Academy of Child and Adolescent Psychiatry (AACAP) clinical practice guideline recommends psychotherapy as the first treatment approach for youth with mild depression.^25^,^26^ The American Psychological Association (APA), in the *APA Clinical Practice Guideline for the Treatment of Depression Across Three Age Cohorts*, and the USPSTF also recommend CBT and interpersonal therapy as preferred psychotherapy interventions for children and adolescents, as both have been supported by a strong body of research as effective treatments for depression, anxiety, and other mental health disorders.^14^,^27^,^28^ The American Academy of Pediatrics clinical practice guidelines *(Guidelines for Adolescent Depression in Primary Care [GLAD-PC]: Part II. Treatment and Ongoing Management)* also are in sync with these psychotherapy recommendations.^29^ Treatment with medications, such as selective serotonin reuptake inhibitors (SSRIs), should be reserved for severe depression or for moderate depression that does not respond to CBT/skills building; these medications should be prescribed in conjunction with counseling that teaches cognitive behavioral skills.^17^ However, it should be noted that children and adolescents with depression respond to antidepressants at a rate of approximately only 50% to 60%.^30^ Relapse rates for MDD are high, ranging from 50% to 85%.^31^ Relapse is likely to occur if the root cause of the depression is a negative or unhelpful pattern of thinking and cognitive-behavioral skills are not learned and practiced.

The AACAP guideline also recommends CBT for patients ages 6 to 18 years with social anxiety, generalized anxiety, separation anxiety, specific phobias, or panic disorder.^32^ For severe anxiety that does not respond to CBT/cognitive-behavioral skills building, SSRIs can be used.^17^,^32^

## The COPE program

The Creating Opportunities for Personal Empowerment (COPE) program was originally developed by this author more than 25 years ago when consulting for a child and adolescent inpatient psychiatric unit in upstate New York. Although the children and teenagers hospitalized on this unit for depression, suicidal behavior, and other mental health conditions had been prescribed numerous psychotropic medications, evidence-based CBT had not been a standard part of their treatment plans. The original iteration of the COPE cognitive-behavioral skills-building program was conceptualized, developed, and evaluated in response to a request for the author to present a weekly health class to the adolescents on the unit as they attended school during the day.

The original COPE program, known as the COPE Healthy Lifestyles TEEN (Thinking, Emotions, Exercise, and Nutrition) program, was designed to teach a combination of cognitive-behavioral skills in 15 weekly sessions (Table 1). The program included all key concepts of CBT (achieving measurable goals, self-monitoring, relationship skills, communication training, cognitive restructuring, problem solving, behavioral activation, relaxation, emotional regulation, and psychoeducation) and also incorporated mindfulness, nutrition, and physical activity, as healthy eating and exercise are key for optimal health and well-being. Evaluation of the program showed very promising outcomes, as levels of depression and anxiety decreased and healthy behaviors increased in the teenagers. Feasibility and acceptability of the program were excellent; the teenagers fully engaged with the content and skills-building activities, practicing them after sessions. The program was manualized into a workbook so that nonpsychiatric providers, including primary care NPs and physicians, social workers, and teachers, could deliver it in addition to psychiatric providers, given the severe shortage of mental health providers across the US.

**Table 1.** Content and skills building in the 15-session COPE Healthy Lifestyles TEEN program

Session #	Session content	Key constructs from the conceptual model and COPE intervention
1	Introduction of the COPE Healthy Lifestyles Pre-TEEN program and goals	Introduction of cognitive-behavioral skills building (CBSB)
2	Healthy lifestyles and the thinking, feeling, behaving triangle	CBSB
3	Self-esteem, positive thinking/self-talk	CBSB
4	Goal setting, problem solving	CBSB
5	Stress and coping	CBSB
6	Emotional and behavioral regulation	CBSB
7	Effective communication; personality and communication styles	CBSB
8	Barriers to goal progression and overcoming barriers; energy balance; ways to increase physical activity and associated benefits	CBSB and physical activity information
9	Heart rate; stretching; physical activity and its positive effects on mental and physical health	Physical activity information
10	Food groups and a healthy body; stoplight diet (red, yellow, and green)	Nutrition information
11	Nutrients to build a healthy body; reading labels; effects of media and advertising on food choices	Nutrition information
12	Portion sizes; “super-size”; influence of feelings on eating	Nutrition information
13	Social eating; strategies for eating during parties, holidays, and vacations	Nutrition information
14	Snacks, eating out	Nutrition information
15	Integration of skills to develop a healthy lifestyle plan; putting it all together	CBSB

∗Twenty minutes of physical activity is included in each session to build beliefs about engaging in regular physical activity.

In the years that followed, further refinements to the 15-session COPE Healthy Lifestyles TEEN program were made and piloted in various groups of adolescents demonstrating excellent outcomes. With large-scale funding from the National Institutes of Health (NIH)/National Institute of Nursing Research, a clinical trial was conducted to test the efficacy of this program with 779 high school students in 11 high schools in the Southwest region of the US. In this clinical trial, adolescents in these high schools were randomized to receive either the COPE Healthy Lifestyles TEEN program or an attention control program that focused on health and safety but did not include any cognitive-behavioral skills building.^33^ Teachers in the high schools that were assigned to deliver COPE attended a 1-day workshop that educated them on how to administer the program to their students and engaged them in practice sessions. The teachers then delivered the 50-minute COPE sessions in their classrooms weekly for 15 weeks. Findings indicated that the teenagers who received the COPE intervention, compared with the teenagers in the attention control group, benefited in terms of multiple outcomes, including an increase in physical activity, decrease in body mass index, improvements in academic competence and health course grades, increase in healthy behaviors, and less alcohol use. For teenagers who began the study with extremely elevated depressive symptoms, teenagers in the COPE program had significantly lower depressive symptoms both immediately after and at 12 months after completing the intervention than the teenagers in the attention control group.^33^,^34^ The 15-session program was rigorously reviewed and selected by the NIH as a research-tested intervention program with the highest dissemination capacity as an adolescent obesity control program.^35^

Because of the positive findings from the 15-session COPE program, a decision was made to separate the 7 CBT-based sessions from the 15-session program and begin testing this 7-session, primarily CBT-based version of COPE with adolescents suffering from depression and anxiety. At the same time, developmentally appropriate versions of the COPE program were created and tested with 7- to 11-year-old children and 18- to 24-year-old young adults. More than 20 studies have supported the various COPE programs' efficacy in decreasing anxiety, depression, stress, and suicidal ideation as well as enhancing self-esteem and healthy lifestyle behaviors in children, teenagers, and young adults.^33^,^34^,^36^-^55^ Reductions in depression and anxiety that have been supported in the numerous experimental studies testing COPE are similar to those achieved by other interventions that have entailed delivery of CBT for a longer duration (for example, 12 to 15 sessions) by mental health care providers.

As in the 15-session COPE program, each session in the 7-session version of the COPE program is followed by skill-building activities that help children and youth to be able to practice what they have learned in COPE in their daily lives (Table 2). “Homework,” otherwise known as skills building in the COPE program, is a necessary component of CBT. In COPE, children, teenagers, and young adults are taught that how they think is related to how they feel and behave. In addition to the other main elements of CBT, COPE teaches children, teenagers, and young adults how to catch, check, and change their unhelpful or automatic negative thoughts in order to feel emotionally better (a process known as cognitive reappraisal). It also teaches them the ABCs (how to monitor for Activating events that tend to cause automatic negative thoughts, and how to turn their unhelpful Beliefs and thoughts around to positive ones to experience healthier Consequences [such as improved mood and healthy behaviors]). Cognitive skills are taught and practiced in COPE. After the skills are learned, they are then used under conditions that trigger a negative thought or affective arousal.

**Table 2.** Content and skills building in the 7-session COPE program

Session #	Session content
1	Thinking, feeling, behaving triangle
2	Self-esteem and positive self-talk
3	Stress and coping
4	Planning, goal setting, and problem solving
5	Dealing with emotions in healthy ways
6	Coping with stressful situations
7	Putting it all together

All COPE programs are manualized into workbooks and can be delivered in 25- to 30-minute individual sessions by nonmental or mental health care providers after completion of a 4-hour online training workshop available at www.cope2thrive.com. As part of this training workshop, the key components of CBT are highlighted. Spanish-language versions of the programs are also available. Thus, COPE provides immediate access to evidence-based interventions for children and youth with depression and/or anxiety who might not otherwise receive any treatment. Until the development of the COPE programs, CBT had traditionally only been delivered by mental health care providers, of whom there is a severe shortage, especially in rural areas throughout the US.

## Adaptation of COPE for other populations

The COPE program has been adapted for adolescents and young adults with chronic daily headaches as well as those who have been bullied with similar positive mental health outcomes.^40^,^56^ In addition, it has been adapted for school-age children with asthma showing improvements in both mental and physical health outcomes.^54^,^55^ COPE also has been adapted for adults as well as for pregnant Black and Hispanic women with depression, anxiety, and stress in an NIH-funded clinical trial and was found to decrease depression and anxiety.^57^

## Reimbursement for COPE in pediatric primary care

The 7-session CBT-based COPE program is being reimbursed in primary care across the country with the Current Procedural Terminology (CPT) code 99214 (established patient office visit, 30-39 minutes), and numerous healthcare providers offer it onsite in their practices, in school-based health clinics, and through telehealth delivery.^47^ A self-delivered online program also is available for adolescents at www.cope2thrive.com. In addition, COPE is being delivered in group and classroom settings in 45- to 50-minute sessions. Primary and secondary schools as well as universities across the country likewise are delivering COPE as a preventive strategy to enhance mental resiliency and prevent mental health disorders.^38^-^40^

To date, COPE has provided immediate access to evidence-based treatment for more than 100,000 children, teenagers, and young adults with depression and anxiety across the nation and globe who might not have otherwise received treatment. Findings from a recent cost analysis indicate a cost savings of $14,262 for every hospitalization for depression that is prevented with COPE, resulting in millions of dollars in cost savings for the US healthcare system.^49^

## NP practice implications

NPs frequently serve as a patient's first touchpoint for addressing mental health concerns in a complex healthcare system. With mounting wait times and mental health care provider shortages, NPs are ideally positioned to manage depression and anxiety successfully when equipped with the appropriate knowledge and skills. NPs should routinely screen for depression and anxiety in children, teenagers, young adults, and adults as recommended by the USPSTF and relevant clinical practice guidelines with tools that are valid and reliable (Table 3) and be prepared to deliver evidence-based intervention programs such as COPE so that affected youth can receive timely treatment. For children and adolescents who have elevated scores on depression and anxiety screening tools, diagnosis should be confirmed through a thorough clinical assessment.

**Table 3.** Valid and reliable screening tools that are free and in the public domain to assess depression and anxiety in children, teenagers, and young adults^58^-^61^

Depression screening tools for adolescents and young adults	PHQ-9	9-item self-report scale
	PHQ-9: Modified for Teens	9-item self-report scale
	PHQ-2	2-item self-report scale
Depression screening tool for children	CES-DC	20-item self-report scale
Anxiety screening tools for adolescents and young adults	GAD-7	7-item self-report scale
	GAD-2	2-item self-report scale
Anxiety screening tool for children and youth 8 to 17 years of age and their parents	SCARED: Child and Parent Versions	41-item self-report scale

Abbreviations: CES-DC, Center for Epidemiological Studies Depression Scale for Children; GAD-2, Generalized Anxiety Disorder-2; GAD-7, Generalized Anxiety Disorder-7; PHQ-2, Patient Health Questionnaire-2; PHQ-9, Patient Health Questionnaire-9; SCARED, Screen for Child Anxiety Related Emotional Disorders

After a diagnosis of depression and/or anxiety is made, intervention with COPE can immediately begin, or the first session can be scheduled, after educating the patient and family about the evidence-based program. Each of the seven COPE sessions should be delivered on a weekly basis. If a week is missed, the next session in the sequence should still be delivered, as each session in COPE builds on the prior session. After the fourth and seventh COPE sessions, it is recommended that depression and anxiety be assessed again with screening tools to document the effects of the intervention. If depression and anxiety are in the moderate to severe ranges and assessment does not reveal a decline in symptoms after the fourth COPE session, strong consideration should be given to starting an SSRI. Children and youth with severe symptoms of depression and reports of suicidal ideation should be transported to the ED for thorough psychiatric evaluation. Those with more severe symptoms of depression and anxiety that are interfering with functioning and not responding to the COPE intervention sessions plus medication should be referred to a psychiatric mental health care provider. Further, all youth and their families should receive education about depression and anxiety, and they should be informed of the 988 Suicide & Crisis Lifeline.^62^ Informative educational handouts for youth with depression and anxiety as well as their parents can be found in the book entitled *A Practical Guide to Child and Adolescent Mental Health Screening, Evidence-based Assessment, Intervention, and Health Promotion, Third Edition*.^17^

A faculty train-the-trainer program is now available and being implemented by NP faculty in schools of nursing throughout the US as a venue for teaching pediatric, family, and psychiatric mental health NP students the key concepts of CBT and providing the necessary education to deliver COPE in their clinical settings. NPs with additional education and clinical experience in child and adolescent mental health care may be eligible for certification as pediatric primary care mental health specialists by the Pediatric Nursing Certification Board.^63^

## Conclusion

Due to a severe shortage of mental health providers across the US, a long lag time exists between the diagnosis of depression and anxiety and the commencement of treatment in children, teenagers, and young adults. COPE is an evidence-based, manualized program for cognitive-behavioral skills building that can be used by NPs, physicians, physician associates/assistants, psychologists, and social workers with reimbursement in pediatric primary care settings, school-based health clinics, and chronic disease specialty clinics to provide immediate treatment for children and other youth suffering from depression and anxiety. The program is designed specifically to shorten this time gap for the pediatric population. An adult version of COPE also is available.

COPE makes screening for depression and anxiety in primary care and chronic disease specialty settings feasible, as this CBT-based program can be offered immediately for those children and adolescents with elevated symptoms. COPE provides access to timely evidence-based treatment for affected individuals who might not otherwise ever receive it. Because of the mental health care professional shortage and the high reoccurrence rate of depression when it is not treated adequately the first time, it is critical to be able to deliver an evidence-based program in nonspecialty settings and to teach youth cognitive-behavioral skills. COPE is an innovative intervention for what is now a major public health epidemic. The program also is being used in primary and secondary schools, universities, community settings, and after-school programs as a preventive intervention to build mental health resiliency skills across the US. It is helping to shift the current healthcare paradigm from one of crisis or sick care to one of wellness, prevention, and early intervention.

Educational training to learn CBT-based skills and deliver the COPE program is available online at www.cope2thrive.com.
